# Surgical and pathological interventions of oropharyngeal and laryngeal disorders in camels (*Camelus dromedarius*)

**DOI:** 10.3389/fvets.2026.1792929

**Published:** 2026-04-21

**Authors:** Fahd AL-Sobayil, Madeh Sadan, Abdulrahman A. Alkheraif, Mohie Haridy

**Affiliations:** 1 Department of Clinical Sciences, College of Veterinary Medicine, Qassim University, Buraydah, Saudi Arabia; 2 Department of Pathology and Laboratory Diagnosis, College of Veterinary Medicine, Qassim University, Buraydah, Saudi Arabia

**Keywords:** animals, diagnostic imaging, osteolipoma, pathology, sialadenitis

## Abstract

**Aim:**

This study aimed to characterize the clinical, hematobiochemical, radiographic, ultrasonographic, laryngoscopic, gross, and histopathological features of oropharyngeal and laryngeal disorders, epulis (osteolipoma and osseous metaplasia), dulla entrapment (tonsillitis and sialadenitis), and obstructive laryngeal mass (mycotic pyogranulomatous laryngitis) and to describe the surgical procedures used for their management in dromedary camels.

**Methods:**

Oropharyngeal and laryngeal lesions in four camels were evaluated through comprehensive clinical, imaging, hematobiochemical, and pathological assessments.

**Results:**

One camel with epulis is presented with a large mandibular mass causing partial oral obstruction, impaired mastication, and swallowing. Radiographs showed a soft tissue density mass on the right cranial mandible. Two camels are presented with dulla entrapment, dysphagia, neck stiffness, and mild respiratory distress. The camel with tonsillitis exhibited dark pinpoint mucosal lesions on the surface of the soft palate with spiny feed impaction; however, the camel with sialadenitis had multiple purulent abscesses in the dulla and submandibular swelling. The fourth camel with obstructive laryngeal mass had a marked respiratory difficulty, and the radiographs revealed a large, irregular soft-tissue mass within the laryngeal lumen. Ultrasonography showed an echogenic, variably anechoic mass, and laryngoscopy confirmed luminal mass obstruction. After surgical removal of these affections in the four camels, histopathology revealed osteolipoma and osseous metaplasia, velar suppurative tonsillitis, suppurative sialadenitis of the minor palatine salivary glands, and mycotic pyogranulomatous laryngitis, respectively.

**Conclusion:**

This study reports four oropharyngeal and laryngeal disorders in camels with obstructive clinical signs. Imaging, laryngoscopy, and histopathology enabled accurate diagnosis, and timely-applied surgery is effective for managing these conditions and rescuing the lives of affected camels.

## Introduction

The importance of camels is obtained from their major roles in producing milk, meat, wool, and leather, as well as their prominence in camel racing within Arab heritage (1–3). Today, they serve as a key source of both meat and milk. Moreover, the general health status of a camel directly influences the volume and quality of its milk and meat, as well as overall production efficiency (1, 2, 4, 5). The gingiva (gum) of the camel is a specialized oral tissue adapted to browsing on coarse, thorny desert vegetation; it exhibits distinctive anatomical and functional features compared with other domestic ruminants (6). Gum disorders in camels (*Camelus dromedarius*) range from mild gingivitis to severe periodontal disease and tumor-like growths in the oral cavity. Recent reports have described maxillary tumors, including ameloblastoma, intraosseous squamous cell carcinoma, and central odontogenic fibroma, emphasizing the clinical significance of oral lesions in this species (7–9). If left untreated, gingival and periodontal lesions can impair mastication, reduce feed intake, cause weight loss, and decrease overall productivity, highlighting the need for early diagnosis and appropriate management (10).

The oropharynx extends from the palatoglossal arches to the base of the epiglottis. Its roof is formed by the soft palate and the palatine diverticulum (11, 12). In camels, the dulla is a downward and forward extension of the soft palate. It is remarkably long, stretching from the caudal margin of the hard palate to a point caudal to the epiglottis, reaching the level of the arytenoid cartilages. The structure of the dulla is more pronounced in adult males than in females, and it commonly protrudes outside the mouth during the rutting season as a sign of sexual behavior (1). Several types of injuries in dulla have been reported in the adult male camels during the rutting season, including protrusions, lacerated wounds, abscesses, hematomas, impaction of feed materials, and gangrene (1, 13). Injuries of the dulla are often caused by pointed or abrasive edges of the cheek teeth and canines, by she-camels during copulation, or by other males during fights. Furthermore, blunt objects, feed materials, and straw can cause such injuries. As a result, an inflammation accompanied by edematous, fluctuating swelling of the dulla may occur, hindering retraction of the dulla into the mouth cavity and progressively worsening the condition. Consequently, these injuries are common reasons for performing the soft palate surgeries in camels (1, 11, 13). Tonsillitis in camels refers to inflammation of the tonsillar tissue. Tonsils serve as a primary immunological barrier in the oropharynx. Anatomical investigations have demonstrated that the lingual and palatine tonsils in camels are well-developed lymphoid structures. They are capable of reacting to ingested and inhaled antigens, making them susceptible to the inflammatory responses when exposed to pathogens or traumatic insults (14). Tonsillitis may occur as a primary condition or alongside other oropharyngeal disorders. Affected animals commonly exhibit dysphagia, excessive salivation, malodorous breath, and varying degrees of respiratory compromise, particularly when tonsillar enlargement partially obstructs the upper airway (15). Tonsillitis has been reported in association with salivary gland disorders and dulla entrapment, suggesting a contributory role in the mechanical and inflammatory airway obstruction. Persistent or untreated cases may adversely affect feed intake, body condition, and productivity (16). Minor salivary glands in camels are widely distributed in the oral mucosa and are essential for lubrication and oral health. These glands may be affected by inflammatory, obstructive, cystic, or neoplastic lesions, including sialadenitis and mucoceles, which can cause swelling, pain, and feeding difficulties if left untreated (17, 18).

The larynx of the camel is a cartilaginous and muscular organ located at the cranial end of the trachea and plays a vital role in respiration, airway protection, and phonation. Anatomical and histological investigations have shown that the camel larynx is composed of the epiglottic, thyroid, cricoid, and paired arytenoid cartilages, supported by intrinsic and extrinsic muscles, and lined by specialized mucosa adapted to respiratory function (19). Laryngeal affections in camels, although infrequently reported, include inflammatory conditions, traumatic or obstructive lesions, and rare mass-forming abnormalities, which may result in respiratory distress, dysphagia, abnormal vocalization, and reduced feeding efficiency. Early clinical evaluation and endoscopic examination are therefore essential for accurate diagnosis and effective management of laryngeal disorders in this species (1, 12, 19–21).

Several oropharyngeal and laryngeal disorders have been documented in earlier studies in different animal species, including sialadenitis in equine (22, 23), canine (24), feline (25), and small (26), and large ruminants (6); palatine tonsillitis in equine (27), canine (28), and small (29) and large ruminants (30); and laryngitis in horses (31), canine (32), and small (33), and large ruminants (34). Osteolipoma has been documented in equine (35), canine (36), and small (37) and large ruminants (38, 39) in various tissues, but it has not been reported in the gingival tissue. Clinical, hematobiochemical, radiographic, ultrasonographic, laryngoscopic, and histopathological evaluations are essential for identifying causes, understanding the pathogenesis, and formulating the appropriate treatment strategies for oropharyngeal and laryngeal disorders. As a result, these conditions are frequently cited as key indications for surgical intervention to achieve a curative outcome (1, 22, 36). Despite the widespread popularity of camels, to the authors’ knowledge, there is no prior research on clinical, hematobiochemical, radiographic, ultrasonographic, laryngoscopic, and histopathological findings or the treatment of oropharyngeal and laryngeal disorders in camels. Therefore, the present study is conducted to describe several oropharyngeal and laryngeal disorders in camels and to evaluate the efficacy of surgical resection as treatment.

## Materials and methods

### Animals and clinical cases

**Figure 1:**
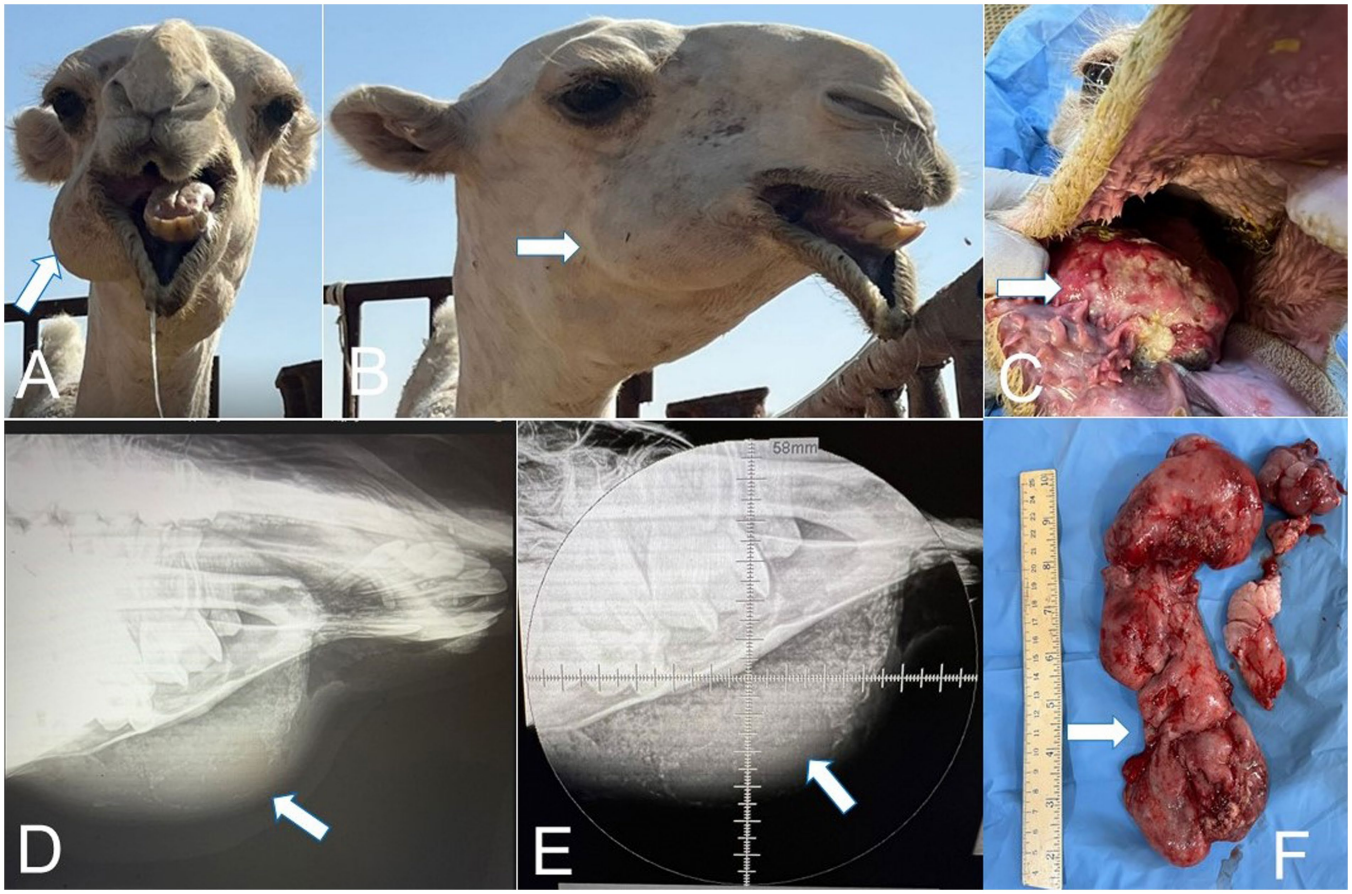
**(A)** Cranial view of a gingival osteolipoma in a camel (arrow). **(B)** Lateral view of a gingival osteolipoma in a camel (arrow). **(C)** Close image of gingival osteolipoma in a camel (arrow). **(D)** Lateral radiograph revealed soft-tissue–density mass located on the lingual (inner) cranial aspect of the right mandible in a camel (arrow). **(E)** Close image of lateral radiograph revealed soft-tissue–density mass located on the lingual (inner) cranial aspect of the right mandible in a camel (arrow). **(F)** Surgically resected gingival osteolipoma in camel (arrow).

Four adult camels (two males, two females) were admitted to the University Veterinary Hospital, Qassim University, Saudi Arabia, in 2025. The camel’s age ranged from 1 to 10 years (mean ± SD: 7 ± 2 years) and weight from 300 to 700 kg (450 ± 120 kg). Breeds of camels were Wadeh (three) and Asfar (one). The camels were selected based on clinical signs, hematobiochemical results, imaging, laryngoscopy, histopathology, and relevant medical history of the oropharyngeal and laryngeal disorders. The latter were documented and categorized by etiology and lesion type (Figure 1). The Committee of Animal Welfare and Ethics at Qassim University, in accordance with the Laboratory Animal Control Guidelines, approved the study protocol (No. 367; March 2025).

### Clinical examination

Clinical examinations were routinely performed on the affected camels. The oral, pharyngeal, and laryngeal cavities were examined physically to describe the conditions: epulis (osteolipoma and osseous metaplasia), tonsillitis, sialadenitis, and obstructive laryngeal mass (mycotic granulomatous laryngitis). The physical characteristics of the injured gums, dulla, and larynx were reported, and the cause, type, and duration of each lesion were determined. The animals’ ages, breeds, and sexes were recorded, and all assessed parameters were compared among the camels examined.

### Hematological and biochemical examination

The hematological and biochemical parameters were examined preoperatively in all camels. EDTA blood samples were analyzed using an automated hematology analyzer (VetScan HM5, Abaxis, Union City, CA, USA) for complete blood counts. Sera were collected, centrifuged, and analyzed with an automated biochemical analyzer (VetScan VS2, Abaxis, Union City, CA, USA) to determine the total protein (TP), albumin (ALB), globulin (GLOB), glucose (GLU), creatinine (CR), creatine kinase (CK), and blood urea nitrogen (BUN); the liver enzymes [aspartate aminotransferase (AST), alanine aminotransferase (ALT), gamma-glutamyl transferase (GGT), and alkaline phosphatase (ALP)]; and the electrolytes and minerals [calcium (Ca), phosphorus (P), magnesium (Mg), sodium (Na), and potassium (K)]. Results were compared with the published reference ranges (4, 39).

### Radiographic, laryngoscopic, and ultrasonographic examinations

Two standard radiographic projections, dorsoventral and lateromedial, were obtained for the affected camels while they were maintained in a sitting position under light sedation (Intravenous/xylazine HCl (2%)/a dose of 0.1 mg/kg). Radiography was performed using a Minx Ray HF 100/30 unit (Toshiba, Tokyo, Japan), and the obtained images were subjectively assessed before surgical intervention. Ultrasonographic examination was subsequently carried out on the sedated camel while placed in a lateral recumbency, using a 3.5–7.5 MHz linear transducer (Aloka, Tokyo, Japan). The ventral cervical region was adequately prepared to allow imaging of the larynx, following the technique described by Sadan et al. (5). Laryngoscopic examination was done after restraining the affected camel in the sternal recumbency under light sedation, with the head elevated, following the procedure described by Nadjmi et al. (40).

### Surgical technique

#### Preoperative medications

Preoperatively, camels received intravenous fluids (0.9% saline 1–3 L and 5% dextrose 1–2 L), intramuscular penicillin–streptomycin (30,000 IU/kg and 10 mg/kg), flunixin meglumine (1.1 mg/kg), and a systemic hemostatic agent (10 mL IM).

#### Surgical management of epulis (osteolipoma)

The affected camel was fasted for 24 h. Under sedation with xylazine (0.3 mg/kg IV) and local infiltration of 2% lignocaine, the mass of epulis was surgically excised (Figure 1) following aseptic preparation (7, 39). Hemorrhage was controlled by applying gauze soaked in tincture of benzoin for 2 minutes, after which the area was dressed in povidone-iodine–impregnated gauze.

#### Surgical management of tonsillitis and sialadenitis

Surgical resection of the affected dulla was performed as definitive treatment to relieve the clinical signs, following comprehensive clinical and hematobiochemical evaluations. After sedation [intravenous xylazine HCl at a dose of 0.3 mg/kg (Norbrook Laboratories, Newry, UK)], the affected camels were secured in sternal recumbency, and the injured dulla was carefully exteriorized using long sponge forceps, combined with gentle digital pressure over the swollen pharyngeal area. The protruded dulla was grasped with a sterile surgical towel and extended to allow proper aseptic preparation, particularly at its base. Local infiltration anesthesia was administered at the base of the dulla using 2% lidocaine hydrochloride (Norbrook Laboratories, UK). Once adequate anesthesia was achieved, overlapping interrupted horizontal mattress sutures were applied to establish hemostasis using coated polyglactin 910 (No. 2) (United Medical Industries Co., Ltd., Riyadh). The injured portion of the dulla was then amputated approximately 3 cm distal to the suture line, and the surgical wound was closed routinely using a simple continuous suture pattern.

#### Surgical management of the laryngeal mass

The laryngeal mass was surgically excised under general anesthesia following a complete clinical and diagnostic evaluation. Anesthesia was induced with xylazine (0.2 mg/kg IV) and ketamine (2–4 mg/kg IV; Zoetis, NJ, USA), intubated, and maintained with 2% isoflurane (Piramal Critical Care Inc., Bethlehem, PA) delivered in 100% oxygen. Via a ventral midline cervical approach, the larynx was exposed, and a longitudinal incision was made through the cricothyroid membrane to access the laryngeal lumen for mass removal (41). The mass was surgically detached and resected at its point of attachment.

#### Postoperative management and follow-up

Postoperatively, antibiotics and anti-inflammatory drugs were administered for 5 days, along with adjunct vitamin AD₃E and meloxicam. Intravenous fluids were provided for 3 days. Camels were stall-confined and monitored daily for 1 month, then discharged after adequate healing. Long-term outcomes were assessed via owner follow-up at 6 months. All animals recovered without complications.

#### Histopathological examination

The excised epulis, dullas, and laryngeal masses were fixed in 10% neutral buffered formalin, paraffin-embedded, sectioned (5 μm), and stained with H&E. Slides were evaluated blindly by a board-certified veterinary pathologist. Special stains (Sudan Black and PAS) were applied to detect lipid cells and fungal hyphae, respectively.

## Results

### Clinical findings

**Figure 2:**
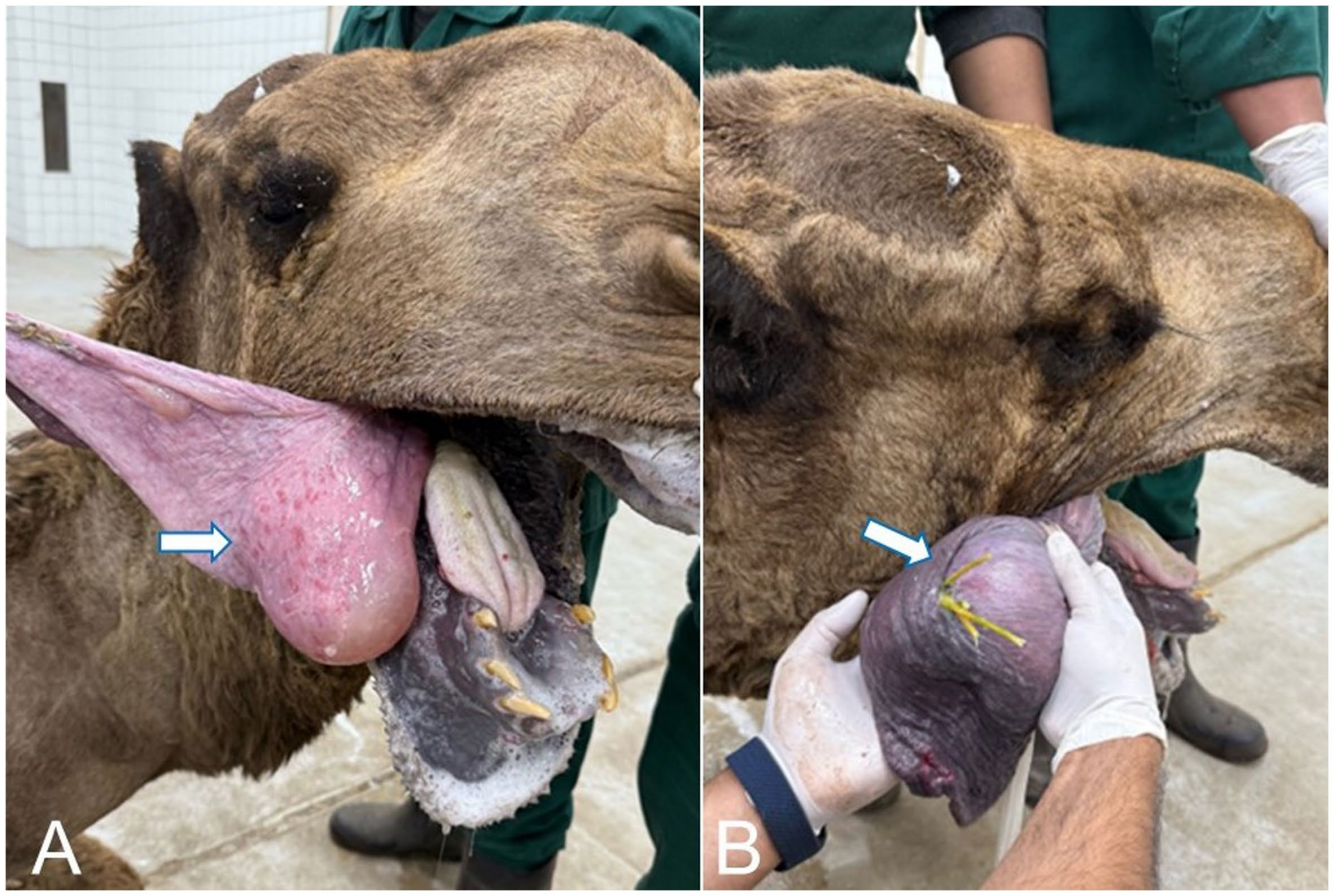
**(A)** Tonsillitis in a camel. Please note the pinpoint dark brown raised foci on the surface of the mucosa of the soft palate (dulla) (arrow). **(B)** Impacted soft palate (dulla) with spiny food materials (arrow).

**Figure 3:**
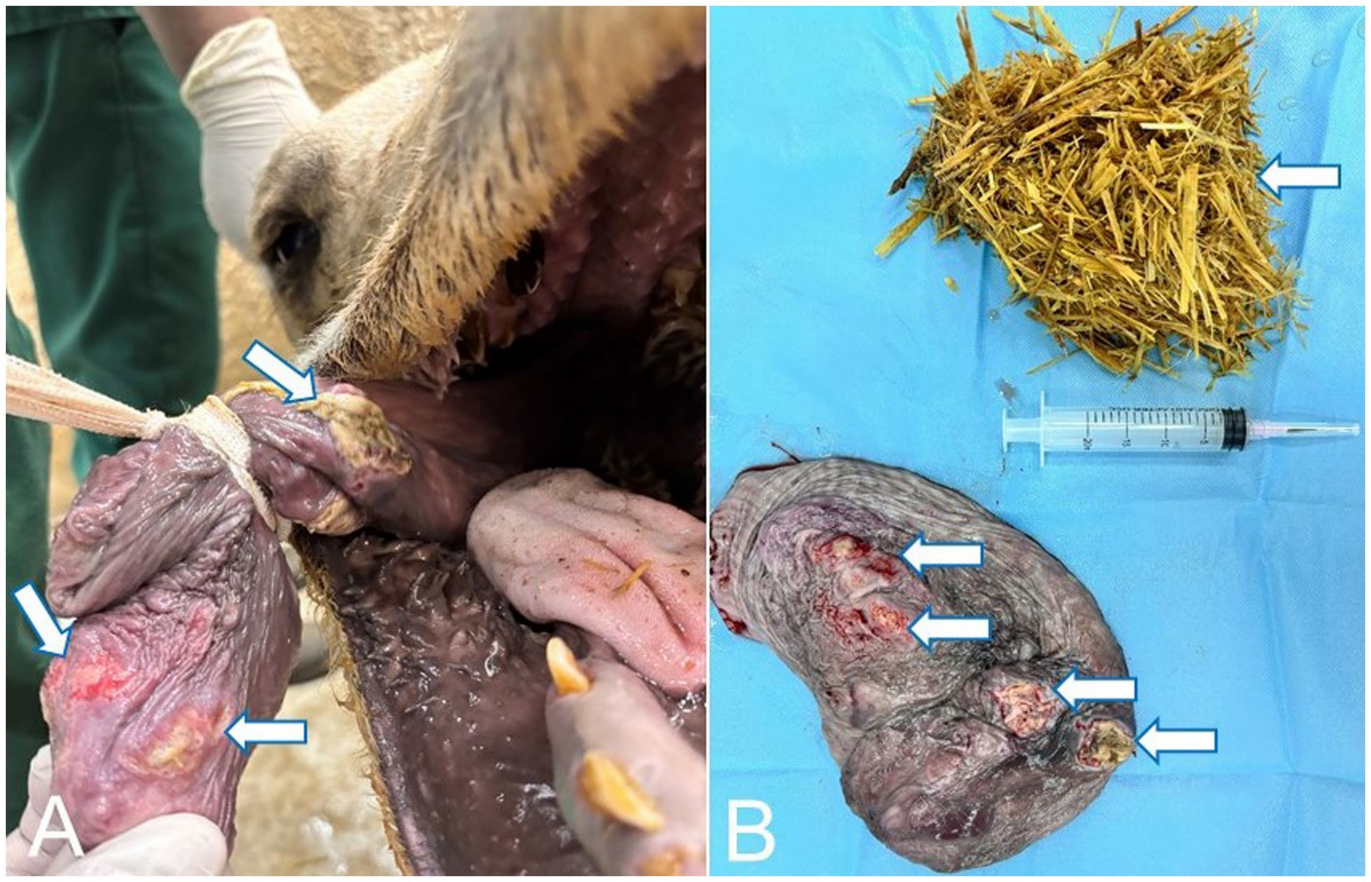
**(A)** Sialadenitis in a camel. Please note the entrapped soft palate filled with multiple abscesses (arrow). **(B)** Impacted soft palate (dulla) with food materials with greenish-yellow pus that opened on the surface, and areas covering the mucosa were ulcerated (arrow).

**Figure 4:**
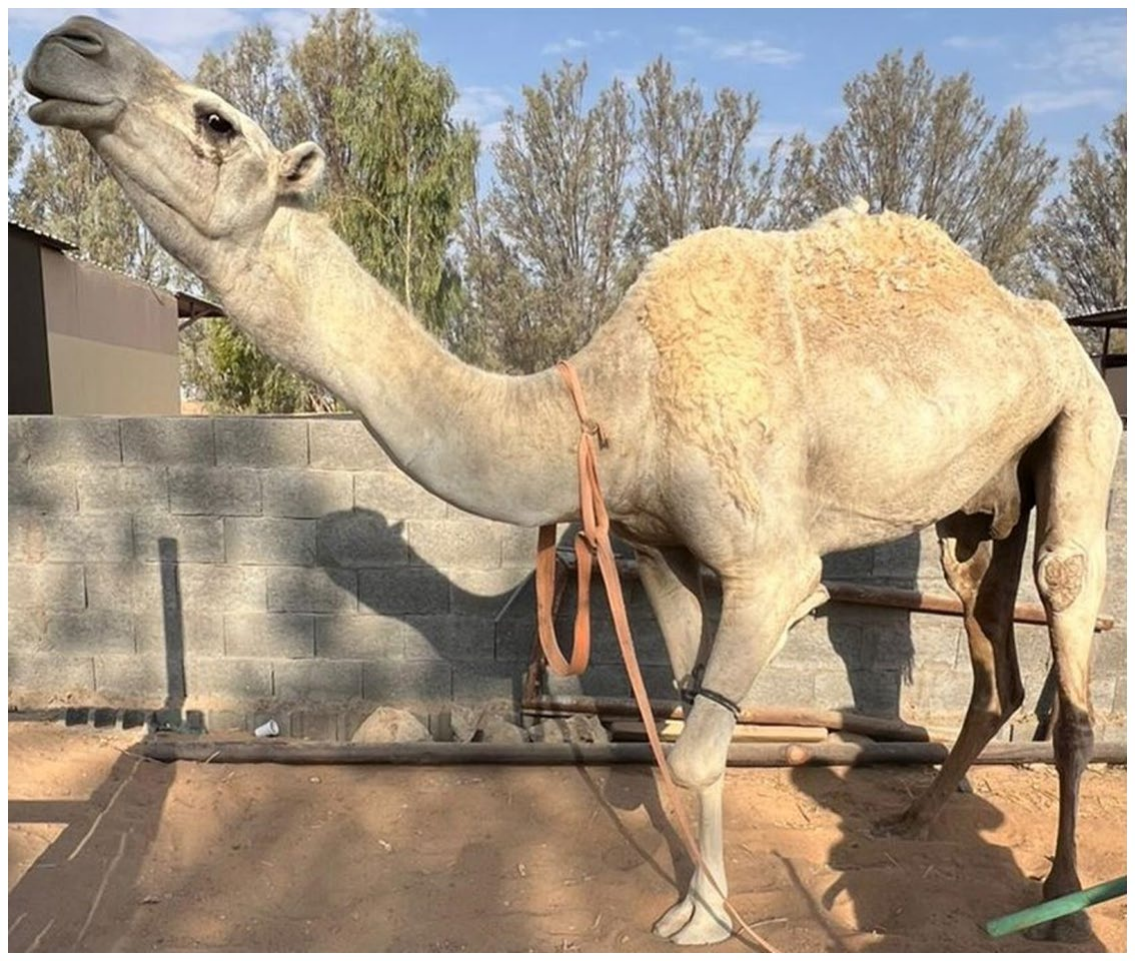
A 7-year-old she-camel presented with a history of partial obstruction of the laryngeal cavity by a large mass (pyogranulomatous laryngitis), which was interfering with normal respiration, causing stiffness, extension of the head and neck, and dyspnea.

**Figure 5:**
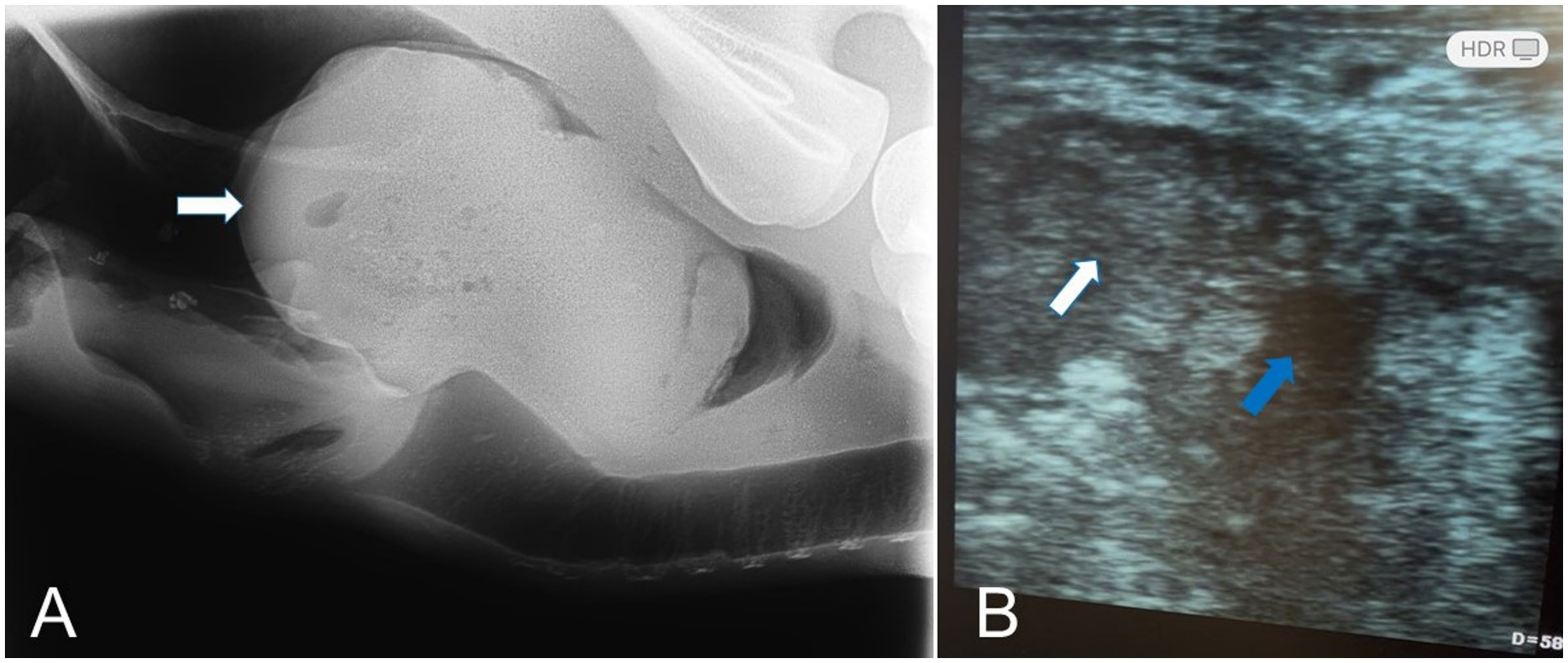
**(A)** A lateral radiograph of pyogranulomatous mycotic laryngitis in a camel revealed a large, irregular soft-tissue mass occupying the laryngeal lumen, outlined by the radiolucent laryngeal air as a negative contrast background (arrow). **(B)** Ultrasonography revealed a sizable, highly vascular echogenic (white arrow) lesion containing variable anechoic areas within the mass (blue arrow).

**Figure 6:**
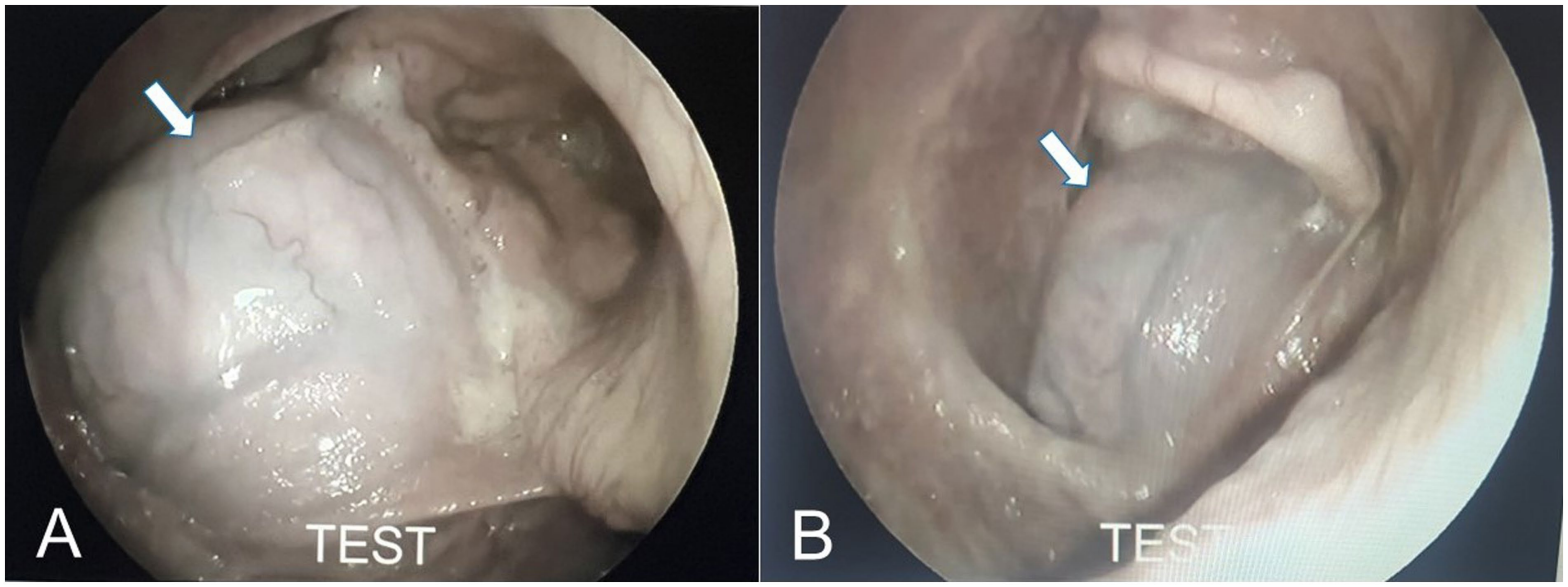
**(A)** Laryngoscopic examination confirmed the pyogranulomatous mass within the laryngeal lumen (arrow). **(B)** Close image of the mass within the laryngeal lumen (arrow).

**Figure 7:**
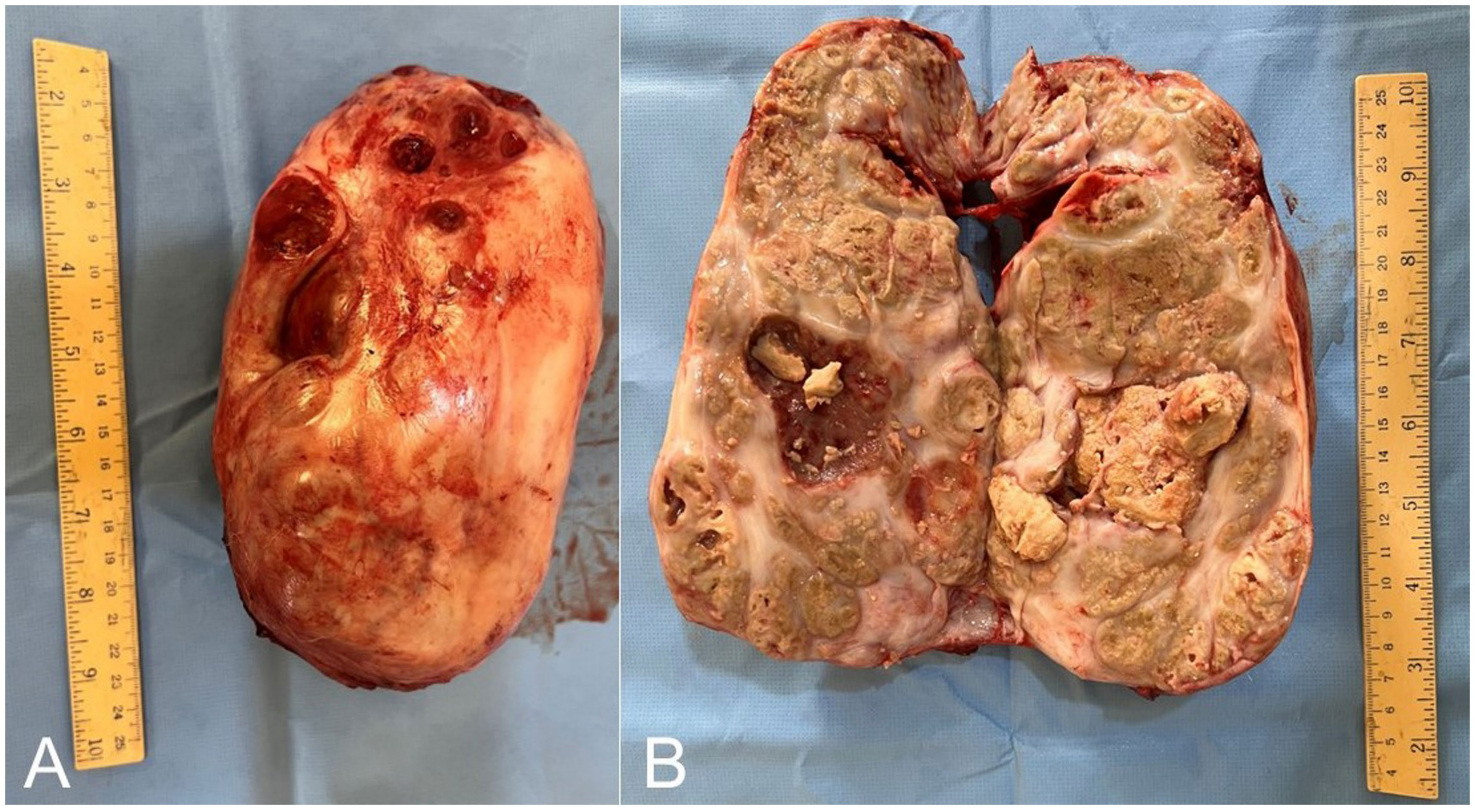
**(A)** The resected laryngeal pyogranulomatous mass. Please note that the large oval mass had a firm consistency, measured 20 cm × 6 cm × 4.4 cm in diameter, and was firmly adherent to the laryngeal tissue. **(B)** The mass is composed of disseminated caseated abscesses that are circumscribed with a tan whitish-gray layer of connective tissue.

This study reported three types of oropharyngeal and one type of laryngeal disorders, namely epulis (osteolipoma and osseous metaplasia), tonsillitis, sialadenitis, and mycotic granulomatous laryngitis (see Figures 1–7; Table 1). A 1-year-old female camel with epulis presented with a history of partial oral cavity obstruction due to a large mass (Figure 1), which was interfering with the normal mastication and causing difficulty in swallowing. The animal appeared restless and had a reduced appetite. Upon oral examination, a large, oblong proliferative mass was observed affecting the lower right gingiva.

**Table 1:** Clinical findings of oropharyngeal and laryngeal disorders in four camels.

Disorder	Age/sex	Main clinical signs	Local findings
Osteolipoma with osseous metaplasia (Epulis)	1-year-old female	Partial oral obstruction, dysphagia, reduced appetite, restlessness	Large oblong proliferative mass on the lower right gingiva
Tonsillitis (Entrapped dulla)	8-year-old male	Dysphagia, neck stiffness, mild respiratory distress	Pin-point dark brown raised foci on the soft palate mucosa, impacted with spiny food material
Sialadenitis (Entrapped dulla)	10-year-old male	Dysphagia, neck stiffness, submandibular swelling, dry mouth	Multiple abscesses in the soft palate containing greenish-yellow pus; ulcerated mucosa
Mycotic granulomatous laryngitis	7-year-old female	Dyspnea, head and neck extension, reduced appetite, restlessness	Large obstructive laryngeal mass

Two male camels (aged 8 and 10 years) with entrapped dullas due to tonsillitis and sialadenitis, respectively, were presented with dysphagia, neck stiffness, and mild respiratory distress. Tonsillitis appeared as pinpoint dark-brown raised foci on the mucosal surface of the soft palate, which was impacted with spiny food materials (Figure 2). Conversely, the camel with sialadenitis (Figure 3) had an entrapped dulla with multiple abscesses, containing greenish-yellow pus that opened on the surface, and multifocal ulcerated mucosa. The camel had a dry mouth, no salivation, and swelling of the submandibular region.

A 7-year-old female camel presented with a history of partial laryngeal cavity obstruction with a large mass. The obstructive laryngeal mass interfered with normal respiration and caused stiffness, extension of the head and neck, and dyspnoea (Figure 4). The animal appeared restless and had a reduced appetite.

### Hematological and biochemical findings

The camel with epulis had reduced red blood cell (RBC) count (6.75 × 10^6^/μL; reference value: 11.3 ± 1.4 × 10^6^/μL), along with decreased hematocrit value (16.78%; normal: 28.9 ± 2.7%) and hemoglobin concentration (14.2 g/dL; normal: 16.0 ± 2.3 g/dL). Conversely, mean corpuscular hemoglobin (MCH) and mean corpuscular hemoglobin concentration (MCHC) were elevated to 21 pg. (normal: 14.7 ± 2.4 pg) and 84.5 g/dL (normal: 57.6 ± 9.0 g/dL), respectively. Biochemical tests revealed an increment in the levels of potassium (5.6 mmol/L; normal: 3.8 ± 0.2 mmol/L), calcium (10.6 mg/dL; normal: 8.6 ± 0.7 mg/dL), and glucose (147 mg/dL; normal: 61 ± 19 mg/dL) and reduction in the levels of sodium (149 mmol/L; normal: 163 ± 2.0 mmol/L), albumin (2.9 g/dL; normal: 4.2 ± 0.4 g/dL), and total proteins (4.6 g/dL; normal: 7.9 ± 0.4 g/dL).

In a camel affected by tonsillitis, the hematobiochemical profile showed several alterations: elevated MCHC (52.5 g/dL) and white blood cell (WBC) count (19.39 × 10^3^/ell) with marked neutrophilia (76.2%), lymphocytopenia (14.5%), and monocytopenia (0.7%). Red cell distribution width (RDW) was elevated (28.6% and RDW 24.2 fly). Glucose was also elevated at 217 mg/dL.

The hematobiochemical profile in camels affected with sialadenitis displayed macrocytosis and hypochromic anemia. The RBC count (8.97 × 10^6^/μL), hemoglobin (15.6 g/dL), and hematocrit (26.63%) were decreased, indicating anemia. Indicators of red blood cell size and hemoglobin content; mean corpuscular volume (MCV) (30 fl), MCH (17.4 pg), and MCHC (58.7 g/dL) were elevated, indicating macrocytosis. RDWc (26% and RDWs 26.6 fl) was also higher than normal. Total WBC were increased (18.94 × 10^3^/μL) with prominent neutrophilia (63.8%), eosinophilia (26.3%), lymphocytopenia (9.3%), and monocytopenia (0.6%). Alkaline phosphatase (ALP) activity was slightly below the reference range (45 U/L), but glucose was elevated (159 mg/dL).

The camel with the laryngeal mass had reduced RBC count (7.29 × 10^6^/μL; reference value: 11.3 ± 1.4 × 10^6^/μL) and hematocrit value (13.12%; normal: 28.9 ± 2.7%). Conversely, eosinophil count (3.96; normal: 2.05 ± 0.57 × 10^3^cell/μL), MCH (18.1 pg.; normal: 14.7 ± 2.4 pg), and MCHC (77 g/dL; normal: 57.6 ± 9.0 g/dL) were elevated. Biochemical analyses revealed increments in the levels of potassium (5 mmol/L; normal: 3.8 ± 0.2 mmol/L), calcium (9.7 mg/dL; normal: 8.6 ± 0.7 mg/dL), and glucose (127 mg/dL; normal: 61 ± 19 mg/dL) and reduction in the levels of creatine kinase (CK) (35 mmol/L; normal: 138 ± 22 U/L), gamma-glutamyl transferase (GGT) (11 mmol/L; normal: 13 ± 5.0 U/L), sodium (147 mmol/L; normal: 163 ± 2.0 mmol/L), and albumin (3 g/dL; normal: 4.2 ± 0.4 g/dL).

### Radiographic, laryngoscopy, and ultrasonographic examinations

Radiographic assessment of the epulis showed a soft-tissue–density mass located on the lingual (inner) cranial aspect of the right mandible (Figures 1D,E) and localized on the gingival soft tissue. The soft tissue mass contained deep radiopaque granules corresponding to osseous tissue and had no continuation with the mandibular bony tissue. Radiographs of the laryngeal mass revealed a large, irregular soft-tissue mass occupying the laryngeal lumen, outlined by the radiolucent laryngeal air as a negative contrast background (Figure 5A). Ultrasonography of the mass demonstrated a sizable, highly vascular echogenic lesion containing variable anechoic areas within the mass (Figure 5B). Also, laryngoscope examination confirmed the mass within the laryngeal lumen (Figures 6A,B). Surgical removal was successfully performed, and the excised tissue appeared as an oval pyogranulomatous mass, had a firm consistency, and was firmly adherent to the laryngeal tissue (Figure 7).

### Gross and histopathological findings

**Figure 8:**
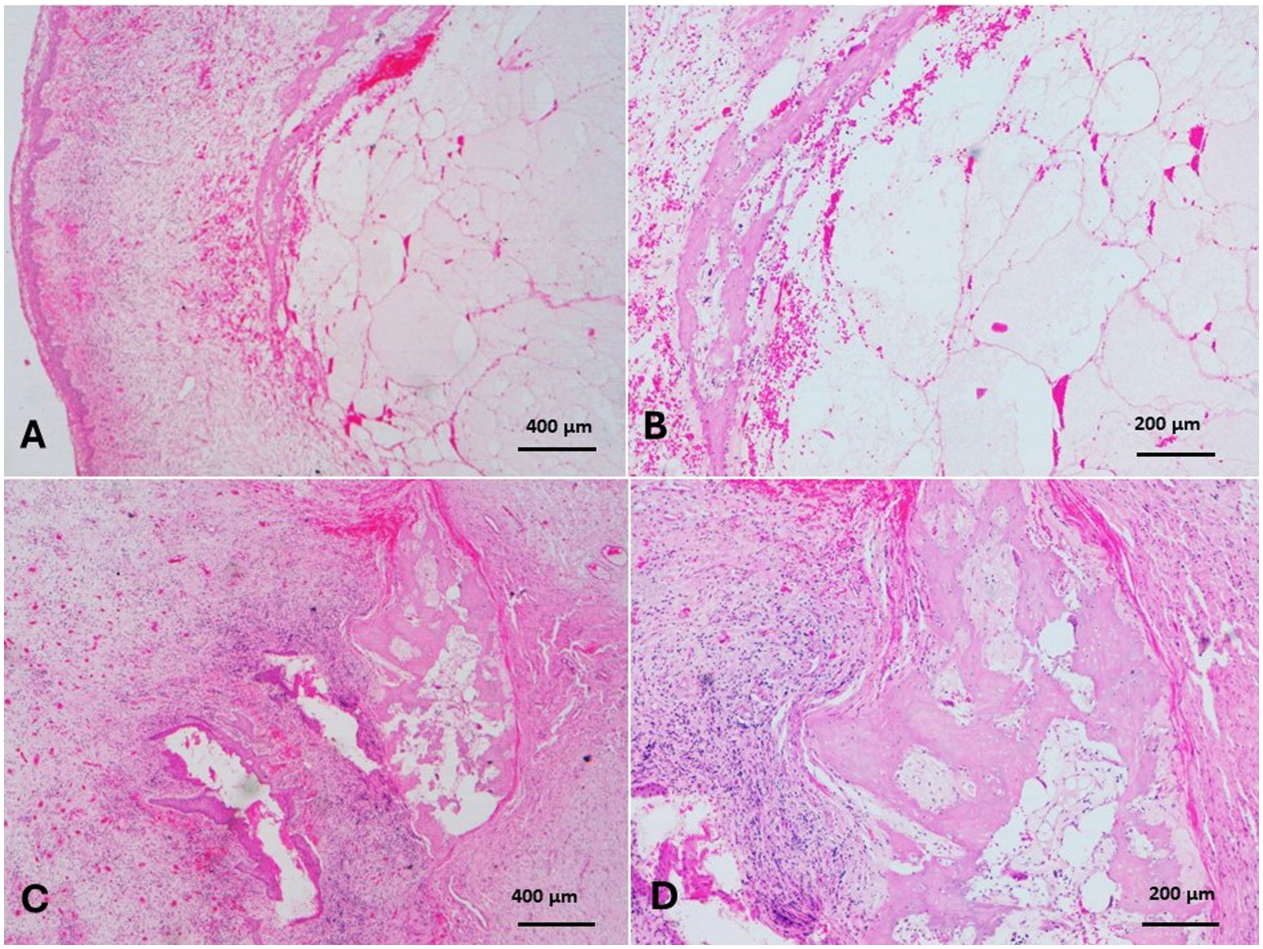
Epulis of camel revealed osteolipoma composed of: **(A)** Large areas of mature fat cells supported by fibrous connective tissue septa (H&E, Bar = 400 μm). **(B)** Focal areas of woven bone surrounded by fusiform mesenchymal cells that circumscribed the lipomatous tissue (H&E, Bar = 200 μm). **(C,D)** Focal areas of osteoid bony tissue (osseous metaplasia) were observed as ectopic osseous tissue containing myeloid tissue in the dense layer of the gum (H&E, **C**: Bar = 400 μm, **D**: Bar = 200 μm).

**Figure 9:**
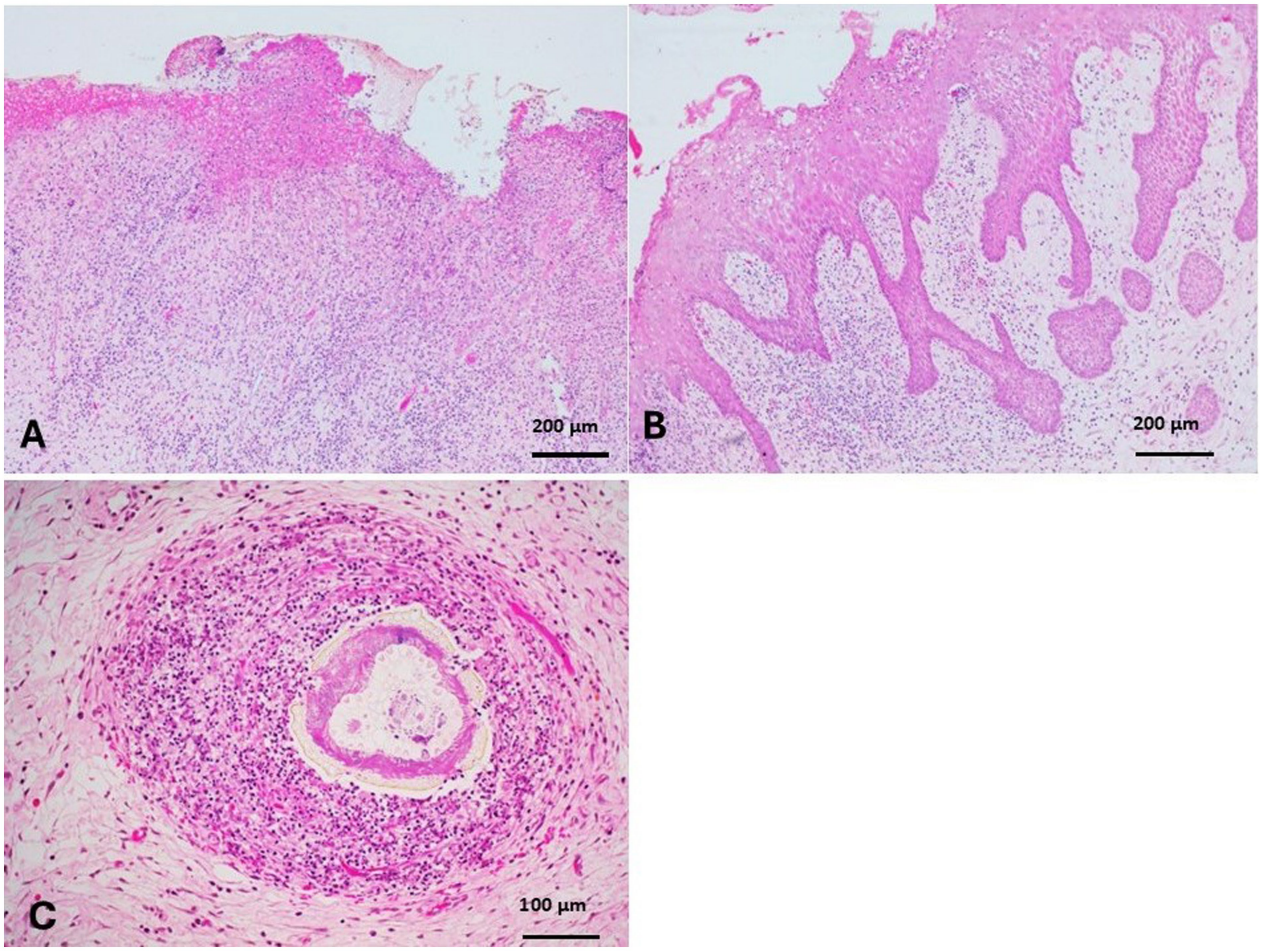
**(A)** The overlying epithelium of the epulis was necrotic and ulcerated, associated with severe neutrophilic infiltration in the superficial layer of the gum (H&E, bar = 200 μm). **(B)** Gingival epithelial hyperplasia was associated with severe inflammatory cell infiltration (H&E, bar = 200 μm). **(C)** Foodstuffs invaded the loose connective tissue layer, leaving a foreign body suppurative reaction (H&E, bar = 100 μm).

Epulis appeared as a large mass on the right cheek that originated from the gum and had no osseous continuation with the mandibular bone (Figure 1C). The mass measured 25× 5× 3.3 cm and was covered with ulcerated and dirty yellowish diphtheritic membrane that penetrated with foodstuffs. The mass was solid and greasy in texture, with a gritty sound in the cut section (Figures 1C,F). Histologically, the epulis mass revealed osteolipoma composed of large areas of mature fat cells, supported by fibrous connective tissue septa (Figures 8A,B), and focal areas of woven bone. Fusiform- mesenchymal cells circumscribed the lesion (Figure 8B). In addition to osteolipoma, focal areas of osteoid bony tissues, as ectopic bone tissue (osseous metaplasia) containing myeloid, were observed in the dense connective tissue layer of the gum (Figures 8C,D). The overlying epithelium was necrotic and ulcerated, and the superficial layer of the gum was severely infiltrated with neutrophils (Figure 9A). Focal gingival epithelial hyperplasia was associated with severe inflammatory cell infiltration (Figure 9B). Foodstuffs invaded the loose connective tissue layer, leaving a foreign body suppurative reaction (Figure 9C).

**Figure 10:**
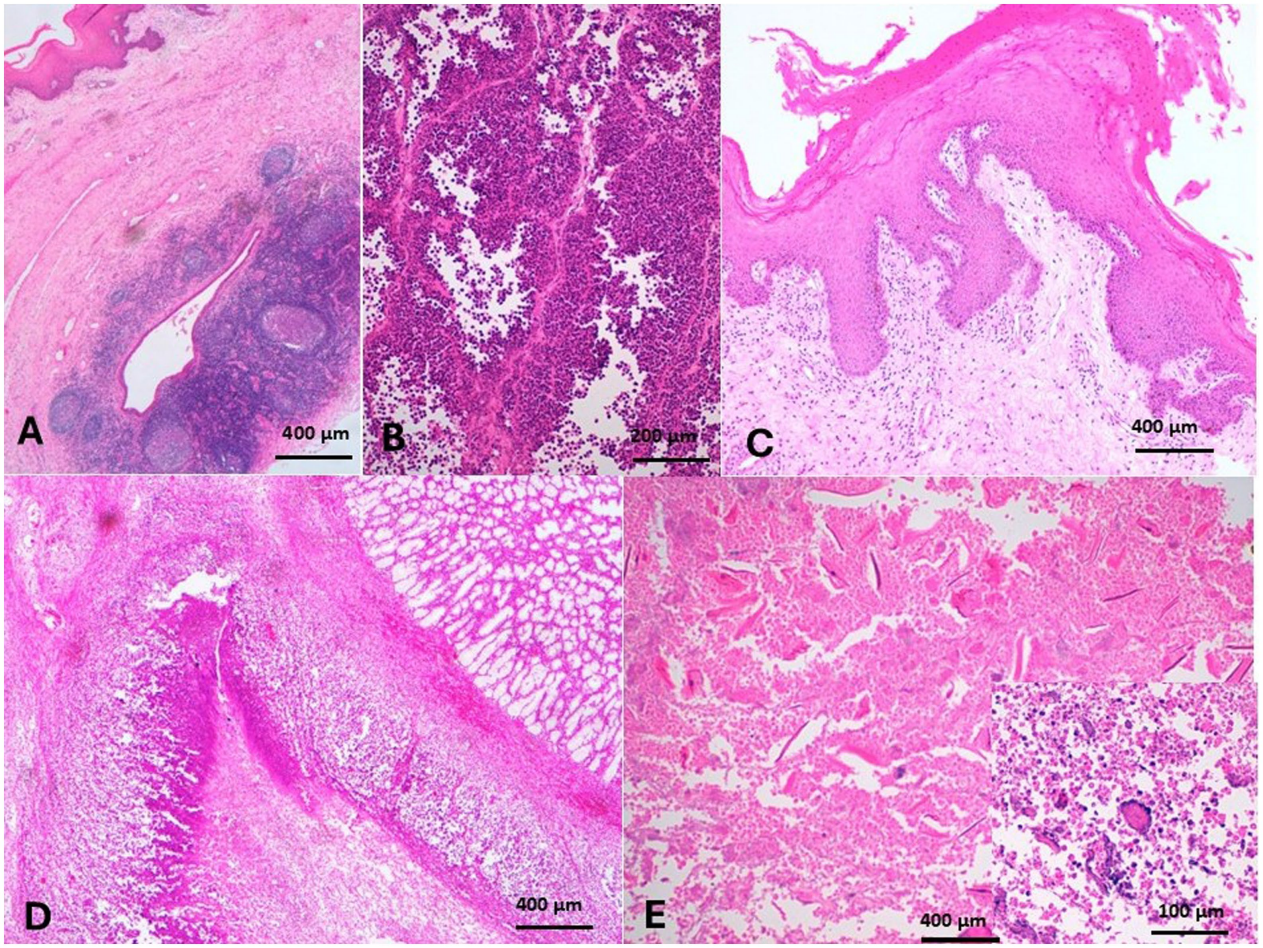
**(A)** The tonsillar nodules were composed of primary and secondary lymphoid follicles separated by parafollicular lymphoid tissue and had crypts that were lined by a non-keratinized stratified squamous epithelium. Some tonsillar follicles were hyperplastic and revealed several germinal centers (H&E, Bar = 400 μm). **(B)** Tonsil nodules revealed suppurative necrosis of the lymphoid follicles, leaving thickened interfollicular dense connective tissue (H&E, Bar = 200 μm). **(C)** The epithelial ridges of the overlying mucosa of the soft palate were hyperplastic, and the submucosa was diffusely infiltrated with neutrophils (H&E, Bar = 400 μm). **(D)** The follicles of the tonsils were also severely necrosed and devoid of lymphocytes associated with necrosis of crypts, leaving an eosinophilic coagulum without structural outlines (H&E, Bar = 400 μm). **(E)** Thorns were observed in the content of massive necrosis and associated with giant cells (inset) and colonies of bacterial infection (H&E, Bar = 400 μm, inset; H&E, Bar = 100 μm).

The soft palate tonsils (velar tonsils) are formed of disseminated nodules with crypts on the oral surface of the soft palate, almost near the junction with the hard palate. Tonsillitis appeared as pinpoint, dark brown raised foci on the mucosa of the soft palate (Figure 2A). The soft palate was swollen, edematous, bright red, and impacted with spiny food materials. Histologically, the normal tonsillar nodules are formed of primary and secondary lymphoid follicles, separated by parafollicular lymphoid tissue, and distributed within the connective tissue of the soft palate, which is covered by stratified squamous keratinized epithelium. The nodule had a crypt, which is lined by a non-keratinized stratified squamous epithelium (Figure 10A). The soft palate tonsillar follicles sometimes appeared hyperplastic and had several germinal centers (Figure 10A). The tonsillar nodules also revealed suppurative necrosis and depletion of the lymphoid follicles leaving thickened interfollicular dense connective tissue (Figure 10B). The lymphoid follicles of the tonsils were also severely necrosed and devoid of lymphocytes associated with necrosis of crypt’s epithelium leaving the tonsillar nodule without structural outlines (Figure 10C). The epithelial ridge of the overlying mucosa of the soft palate was hyperplastic. The submucosa was diffusely infiltrated with neutrophils (Figure 10D). Massive necrosis of the tonsils induced a pressure atrophy of the salivary glands due to surrounding fibrosis. Thorns were observed in the area of massive necrosis, along with giant cells and colonies of bacterial infection (Figure 10E).

**Figure 11:**
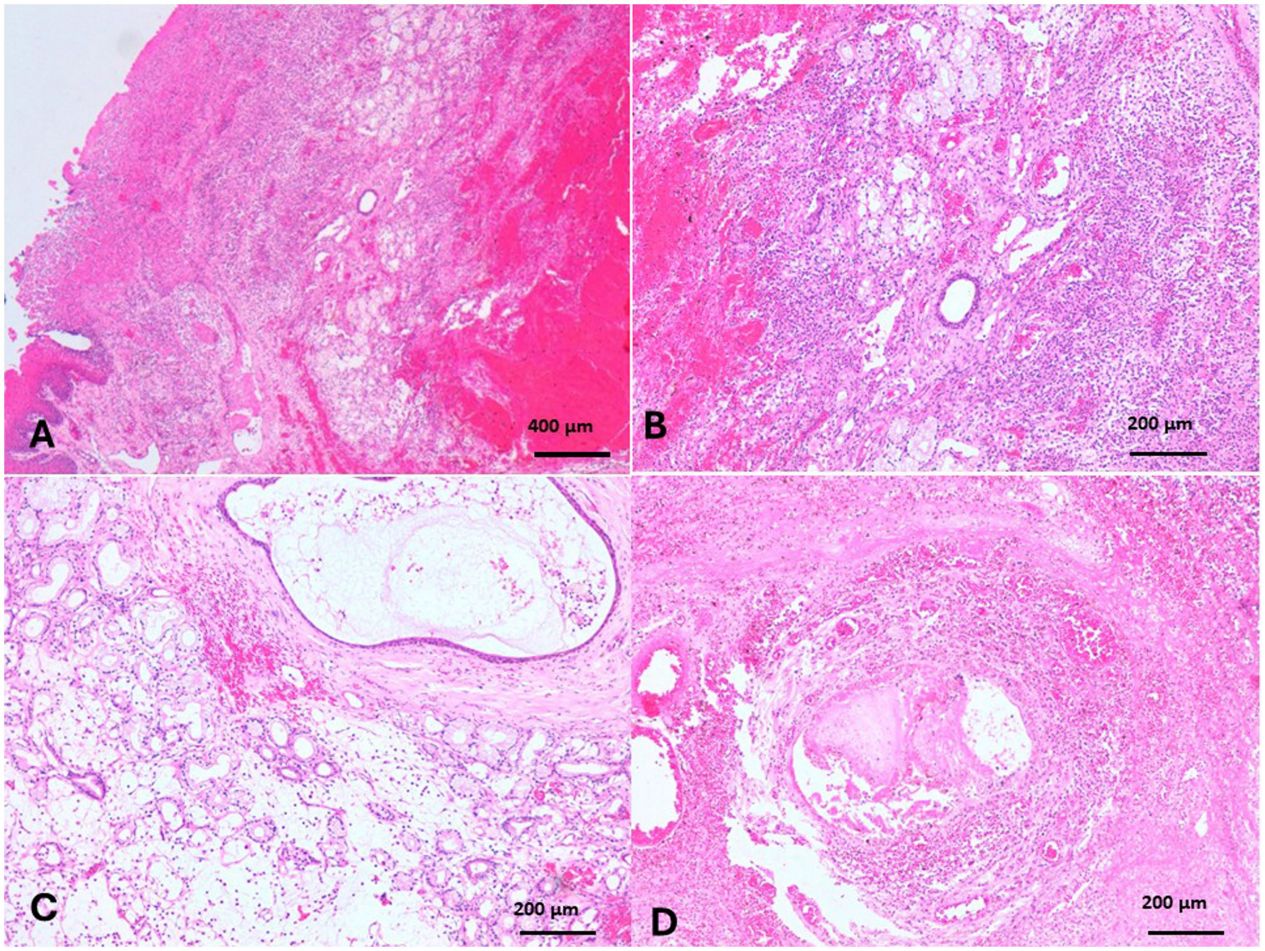
Sialadenitis of minor salivary glands of soft palate revealed **(A)** hemorrhages and multiple abscesses in the submucosa, and the overlying mucosa were ulcerated and healed with granulation tissue (H&E, Bar = 400 μm). **(B)** Destruction, necrosis, and fibrosis of salivary glands (H&E, Bar = 200 μm), and **(C)** periductal fibrosis (H&E, Bar = 200 μm) were detected in the tissue surrounding the abscesses. **(D)** Ductal obstruction with exudate, organized periductal fibrosis, and the surrounding glandular tissues revealed liquefactive necrosis were noticed (H&E, Bar = 200 μm).

The minor palatine salivary glands, branched tubule-alveolar mucous and seromucous glands, are located within the propria and submucosa in the hard palate and increase toward the apex of the soft palate. The camel with sialadenitis grossly revealed multiple abscesses of dulla, contained greenish-yellow pus that opened on the surface, and multifocal areas of the covering mucosa were ulcerated. Histologically, hemorrhages and multiple abscesses were observed in the submucosa of the affected soft palate. The overlying mucosa was ulcerated and healed with granulation tissue (Figure 11A). Destruction, necrosis, and fibrosis of the salivary glands (Figures 11A–C) and periductal fibrosis (Figure 11C) were observed in the tissue surrounding the large abscesses. Ductal obstruction with exudate encircled with organized connective tissue, where the surrounding glandular tissues had a liquefactive necrosis (Figure 11D).

**Figure 12:**
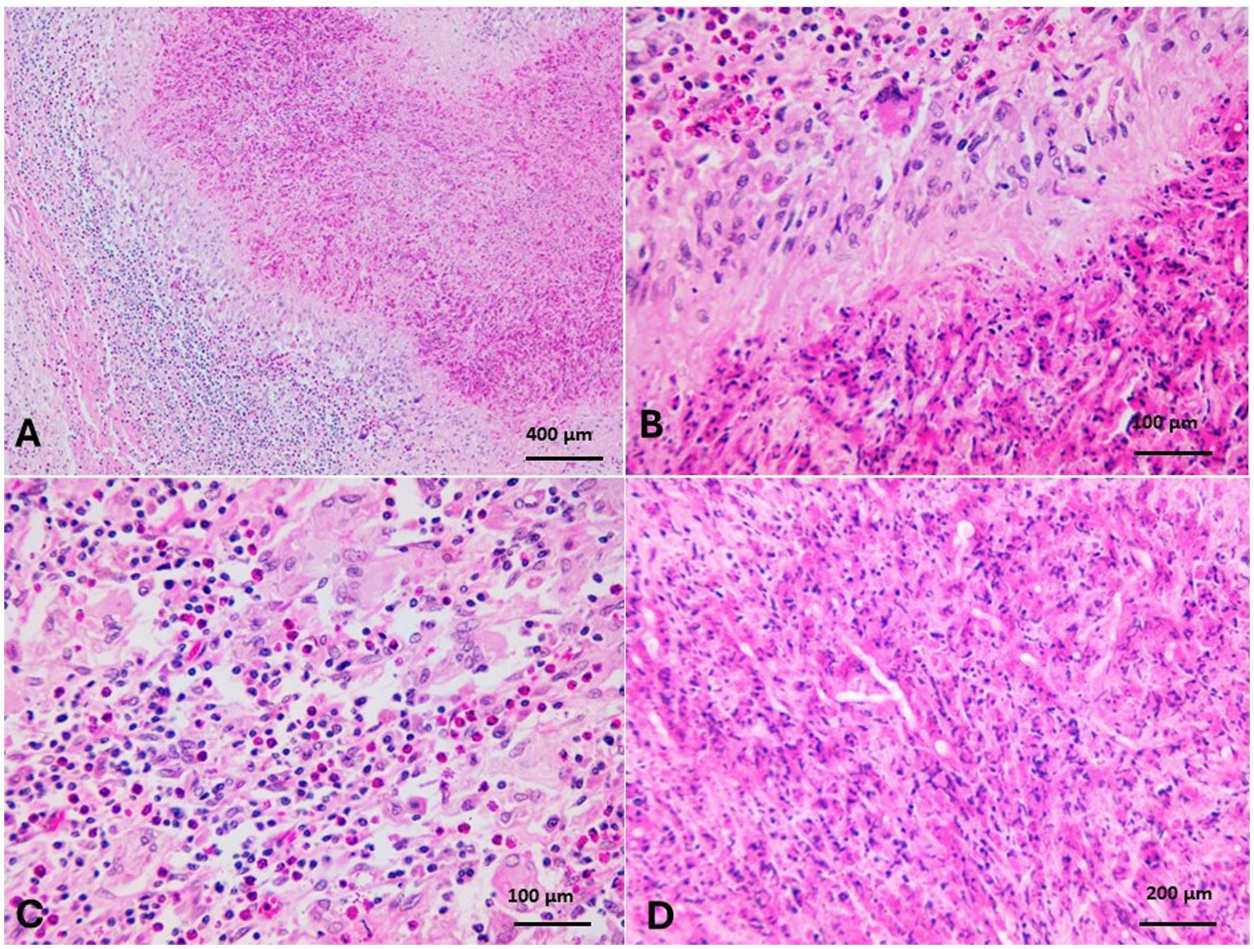
**(A)** Mycotic granulomatous laryngitis with a necrotic eosinophilic center containing cellular debris rich in eosinophils and fungal hyphae, surrounded by macrophages, epithelioid cells, giant cells, and rich eosinophilic infiltration (H&E, Bar = 400 μm). **(B)** The necrotic centers were surrounded by a fibrous layer rich with eosinophilic infiltrate and giant cells (H&E, Bar = 100 μm). **(C)** The tissues separated the pyogranulomatous lesions, which were rich in neutrophils, eosinophils, macrophages, and giant-cell infiltration (H&E, Bar = 100 μm). **(D)** Hyphae had parallel cell walls, distinct septa, and a dichotomous branching pattern (*Aspergillus* hyphae) (H&E, Bar = 200 μm).

**Figure 13:**
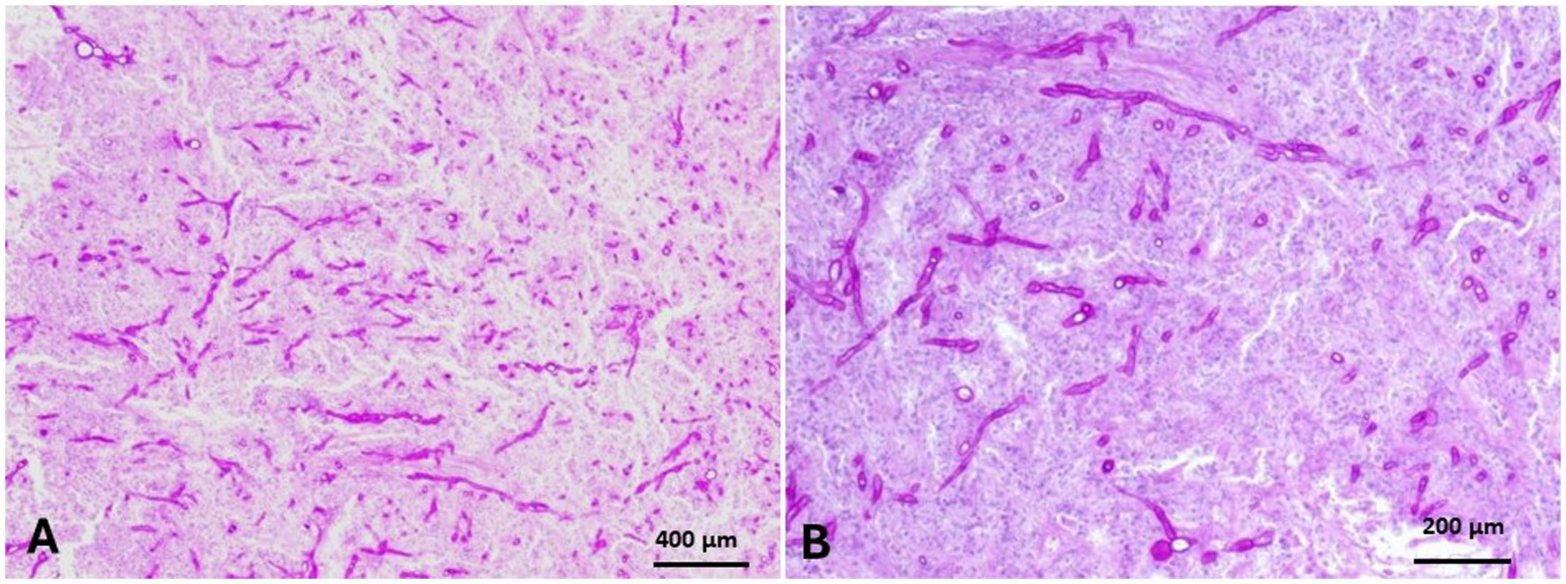
**(A)** Fungal elements revealed hyphae that were characterized by parallel cell walls, distinct septa, and a dichotomous branching pattern (*Aspergillus* spp.) (PAS, bar = 400 μm). **(B)** Fungal hyphae were present in granulomatous lesions and invaded adjacent structures (PAS, bar = 200 μm).

Grossly, the laryngeal mass was large oval mass that had a firm consistency, measured 20 cm x 6 cm x 4.4 cm in diameter and was firmly adherent to the laryngeal tissue (Figure 7A). The mass composed of disseminated caseated abscesses that circumscribed with tan whitish gray layer of connective (pyogranulomatous lesions) (Figure 7B). The histopathology of the mass revealed mycotic granulomatous laryngitis with a necrotic eosinophilic center, containing cellular debris rich of eosinophils and fungal hyphae (Figure 12D). It surrounded by a cellular zone composed of macrophages, epithelioid, giant cells, and rich eosinophils (Figures 12A,B). The cellular zones were surrounded by a fibrous layer rich with eosinophilic infiltrate and giant cells (Figure 12B). The interstitium between the pyogranuloatous lesions was rich in neutrophlic, eosinophilic, macrophages and giant cell infiltration (Figure 12C). To improve the visualization of the fungal elements, periodic acid-Schiff was applied, and the pyogranulomatous lesions revealed plenty of hyphae that characterized by parallel cell walls, distinct septa, and a dichotomous branching pattern (Figures 12D, 13A,B). These morphological characteristics are in compromise with *Aspergillus* Spp. Fungal hyphae were present mainly in the pyogranulomatous lesions and also invaded adjacent structures.

### Treatment outcomes

A strong correlation was reported between the duration of intervention for the oropharyngeal and laryngeal disorders and the general health conditions of the affected camels. Favorable outcomes were associated with early diagnosis, prompt treatment, and diligent post-operative management. Follow-up assessments were conducted over a period of 6 months through phone communication and field visits with camel owners. The effect of surgical resection of osteolipoma, entrapped dullas, and mycotic laryngeal mass on the ability to eat, swallow, and breathe of the affected camels, and if any abnormal surgical-site discharge, and the overall functional recovery parameters were evaluated. All camels showed full restoration of normal function within 6 months after surgery.

## Discussion

Injuries involving the oropharyngeal and laryngeal lumen are serious conditions in camels and often necessitate surgical intervention to manage their associated complications. However, the literature provides limited information regarding the clinical presentation, hematobiochemical alterations, imaging characteristics, histopathological features, and the therapeutic approaches for these lesions. Therefore, detailed clinical, laboratory, radiographic, ultrasonographic, laryngoscopic, and histopathological findings of the oropharyngeal and laryngeal disorders were reported, and the surgical outcomes in treating these conditions in camels were highlighted. Three types of oropharyngeal and one type of laryngeal disorders, namely, epulis (osteolipoma), tonsillitis, sialadenitis, and mycotic pyogranulomatous laryngitis, respectively, were reported.

Clinical examination is routinely used to diagnose the oropharyngeal and laryngeal disorders in camels. In the present study, a camel with an osteolipoma had a history of partial obstruction of the oral cavity due to a large mass that interfered with normal mastication and caused difficulty in swallowing. These observations were consistent with those described in earlier reports (35–39). The camels with entrapped dulla, due to tonsillitis and sialadenitis, had dysphagia, neck stiffness, and mild respiratory distress. These clinical findings are similar to the findings reported by Sadan and El-Shafaey (1). The camel affected with pyogranulomatous laryngitis had a partial obstruction of the laryngeal lumen, which impaired normal respiration and resulted in neck and head extension, stiffness, and noticeable dyspnea. The affected camel showed signs of restlessness and a diminished appetite (33, 34).

The hematological examination of the camel with osteolipoma revealed anemia, which could be due to the bleeding accompanying the osteolipoma and recurrent traumatization. Elevated potassium levels, along with decreased levels of sodium, albumin, and total protein, indicated anorexia, dehydration, and inflammatory responses associated with the osteolipoma. The increase in glucose level could be related to the mobilization of fat stores as a glucose source, particularly under fasting conditions or prolonged inflammation in camels (5, 42, 43). In a camel affected by tonsillitis, the hematobiochemical changes are consistent with systemic inflammation and stress. The MCHC was elevated, reflecting altered erythrocyte hemoglobin content compared with established reference ranges in healthy camels (5, 42–44). The elevated WBC count with marked neutrophilia and concurrent reductions in lymphocyte and monocyte percentages indicated a pronounced inflammatory leukogram typical of the infectious conditions in camels. RDW was also elevated, suggesting increased variability in erythrocyte size. Furthermore, the glucose level was elevated due to stress-related hyperglycemia, which is commonly observed during acute disease in camels. These alterations are aligned with the pathological inflammatory findings (5, 42, 43).

In camels with sialadenitis, the hematobiochemical alterations are consistent with systemic inflammation and stress, including mild anemia, macrocytosis and anisocytosis, leukocytosis, neutrophilia, eosinophilia, lymphocytopenia, and monocytopenia, as they are commonly reported in inflammatory conditions in camels (5, 42, 43).

The camel affected by mycotic pyogranulomatous laryngitis demonstrated a reduction in red blood cell count and hematocrit values, but an increase in eosinophil percentage, MCH, and MCHC. Increased levels of potassium, calcium, and glucose and decreased levels of CK, GGT, sodium, and albumin were also reported. These alterations may reflect the reduced feed intake, respiratory compromise, and the prolonged inflammatory nature of the condition. Similar findings were reported in chronic granulomatous and mycotic disorders in camels and other large ruminants (6, 45–47).

The radiographic and ultrasonographic findings in the present study provide valuable insights into the diagnostic characteristics of soft-tissue masses affecting the oropharyngeal and laryngeal regions of camels. In osteolipoma, the identification of a well-defined soft-tissue mass containing discrete radiopaque foci without continuity with the mandibular bone supports its origin from gingival soft tissues rather than the mandibular framework. Similar radiographic features have been described in osteolipomatous and mineralizing soft-tissue lesions in large animals (6, 45). In pyogranulomatous mycotic laryngitis, the radiographs revealed an irregular intraluminal soft-tissue mass outlined by the laryngeal air column, a negative contrast effect commonly reported in obstructive laryngeal lesions (6, 48). Ultrasonography demonstrated a vascular echogenic mass with mixed anechoic areas, which is consistent with chronic granulomatous inflammatory lesions in ruminants and camelids (45, 48). Laryngoscopic examination provided a definitive confirmation of the intraluminal obstruction and is considered a critical diagnostic modality for upper airway diseases in large animals (6). The successful surgical excision of these obstructive masses highlighted the importance of integrating multimodal imaging with endoscopic evaluation to achieve accurate diagnosis and to guide effective management, as recommended in earlier camelid and ruminant clinical reports (48).

The gum of camels is histologically composed of a covering mucosa, loose superficial, and dense deep connective tissue layers. Oral disorders in camels at Maiduguri Abattoir, Borno State, Nigeria were reported in 313 heads of camels, comprising 114 (41.67%) males and 199 (58.33%) females. The prevalence rate of the disorders observed include dental tartar and calculus (5.42%), inward rotation of incisors (2.87%), fractured teeth (7.66%), maleruption (0.31%), oligodontia (0.31%), gingivitis (4.15%), ulcerated cheek (0.63%), and presence of foreign body (0.31%) in the mouth. About 22.61% of the total heads had one disorder or another; 7.98 and 14.06% of the disorders were among male and female heads, respectively (49). Three cases of tooth root abscesses in camels (Old World Camelids) were reported (50). Four camels had maxillary masses that appeared as multicystic and expanded the maxillary bone. The tumors were diagnosed by histopathologic examination as conventional ameloblastoma, two cases as intraosseous squamous cell carcinoma, and central odontogenic fibroma with ossification (7). A fibrous epulis (1.2 cm in diameter) has been recorded in a one-humped camel on the labial part of the upper gum (51). Whorls and bundles of fibroblasts and fibrocytes were the main components of the epulis, with two keratin foci centrally located. Odontogenic tumors have been recorded in camels, characterized by odontogenic epithelium and mesenchyme, and they originate from sites of teeth in the mandibular and maxillary bone (7). The present case was neither fibromatous epulis nor an odontogenic tumor, as the former contained collagenous fibers and fibroblastic proliferation, whereas the latter contained odontogenic epithelium, which was absent in osteolipoma and osseous metaplasia. Osteolipoma has been observed very rarely in humans (less than 1% of mesenchymal lipomas). Only 20 cases of oral osteolipoma have been reported in humans based on 69 searches in the PubMed database (52). To the authors’ knowledge, oral or gingival osteolipoma has never been recorded in veterinary medicine. Oral fibrolipoma has been recorded in 112 dogs in a multi-institutional retrospective compilation of cases submitted to diagnostic pathology services, which are characterized histologically by a typical lipoma with variable amounts of paucicellular and collagenous fibrous components but devoid of osseous tissue (53). Osteolipoma, characterized by mature yellow adipocytes and residual bony trabeculae, has been recorded in the ribs of a sheep (37) and a Leonberger (54) named intraosseous lipoma (37). A lipoma with marked calcified osseous tissue and sometimes fat cell necrosis is diagnosed as osteolipoma, osseous lipoma, ossifying lipoma, or lipoma with osseous metaplasia (55, 56). An intracranial ossifying lipoma was recorded in a juvenile pig (57) and a laboratory rat (58), characterized by lobules of adipose tissue interrupted by fibrous connective tissue septa, an outer layer of well-differentiated cortical bone, and an inner core of adipose tissue and hematopoietic cells (57). Two cases of osteolipoma in canine skin were reported with findings similar to those of the present study (56). Osseous metaplasia is the formation of bone in abnormal locations in which fibroblast-like cells differentiate into osteoblasts and osteoid formation. Abnormal locations of bone formation in dogs commonly include mammary tumors and salivary mucocele (59), as well as other tissues such as eyes, skin, lateral abdominal wall, and the external auditory canal (60). In camels, osseous and osteo-lipomatous metaplasia were recorded in the liver (61, 62) and metastatic gastric adenocarcinoma (63). Apart from osteolipoma in the present case, focal areas of osseous metaplasia of the dense fibrous layer of the gum were observed. This is the first record of osseous metaplasia in gingivitis in camel and veterinary medicine.

A few papers studied the anatomy and histology of camel tonsils. A camel has six types of tonsils. They are well-developed and visible, particularly the palatine one. The lingual, palatine, velar, and paraepiglottic tonsils are arranged into closely assembled lymphoid follicles and show a multitude of crypt openings into the oropharyngeal tube. However, the nasopharyngeal tonsils (pharyngeal and tubal) include loosely connected follicles that extend into the overlaying epithelium (14, 64). There are no previous reports on tonsillitis in camels, but it is common in dogs and cats (65). Suppurative nasopharyngeal tonsillitis and palatine tonsillar cryptitis were recorded in calves infected with *Mycoplasma bovis*, and the histopathological picture revealed degenerative and necrotic changes of the cryptic epithelium and lymphoid follicles. The lumen of these tonsillar crypts was occluded by necrotic cellular debris, resulting in microabscesses (66). The present case of camel also presented with suppurative tonsillitis in the velar tonsils. The foodstuff penetrating the soft palate was observed grossly and histopathologically, which might incite inflammation. Foreign body giant cells, bacterial colonies, and thorns were observed in the soft palate tissue. Actinomyces -associated plant thorns are implicated in the deep stomatitis in animals (67). The major salivary glands of camels are the parotids, mandibular, and sublingual salivary glands (68); however, the minor salivary glands are the labial, buccal, palatine, von Ebner’s glands, and lingual salivary glands (18). The palatine salivary glands were in the caudal part of the hard palate and in the entire length of the soft palate. The palatine glands were most numerous at the apex of the soft palate (18). Sialadenitis in camels has not been recorded except as part of rabies infection. Moreover, sialadenitis might be a sequel to sialolithiasis. Sialolithiasis has been recorded in camels and other farm animals (17, 69, 70). The present case of sialadenitis did not suffer from sialolithiasis. The sialadenitis in the present case might be due to a bacterial infection of the soft palate, leading to entrapment during expulsion. Several abscesses, hemorrhage, and ulceration of the mucosa of the soft palate were detected, suggesting that palatine sialadenitis is a primary condition concurrent with inflammation of the soft palate (dulla). Inflammation of minor salivary glands in dogs has been reported and may be of non-specific cause (71).

Laryngitis is not as common a primary lesion but usually an extension from the upper or lower respiratory tract, except in cats (72). A search of the PubMed database revealed records of diphtheroid necrotic inflammation of the larynx in calves and cattle (73, 74). Necrotizing stomatitis, glossitis, pharyngitis, laryngitis, ruminitis, and liver abscesses and thrombosis of the vena cava, as well as interdigital foot rot in cattle, are commonly associated with *Fusobacterium necrophorum subsp. necrophorum* (75). Laryngitis in camelids was recorded only once, with laryngeal abscessation (not involving the cartilage) in a 10-day-old alpaca, and *Mannheimia hemolytica* was isolated from the case (76). In the present case, mycotic pyogranulomatous laryngitis with morphological features of fungal hyphae, in the context of aspergillosis, was diagnosed. Mycotic laryngitis is common in immunocompromised human patients, with a higher prevalence of *Aspergillus* infection (77). The camel in the present case was in good body condition and had no signs of starvation or immunosuppression. *Aspergillus* spp. are saprophytic filamentous fungi commonly found in soil, decaying vegetation, and on seeds and grains, with occasional potential to infect living animal hosts (78). Fungal spores could gain entry into the deep tissue of the tongue, pharyngeal or laryngeal mucosa, producing deep pyogranulomatous lesions such as wooden tongue disease (79). So, the fungus might enter the deep layer of submucosa of the larynx through penetration by foodstuffs that proceed to the mycotic pyogranulomas. Bronchopulmonary and disseminated infections are major forms of Aspergillosis in dogs and cats (80). *Aspergillus* species causes mycotic pneumonia, gastroenteritis, mastitis, placentitis, and abortions in ruminants and guttural pouch infections, keratomycosis, and pneumonia in horses (81). There are no lesions in the present case except mycotic laryngitis.

The present work has several limitations. The small number of cases limits generalizability and precludes statistical analysis or determination of prevalence and risk factors. Furthermore, part of the follow-up evaluation depended on the owner’s reports, introducing potential subjective bias. Practical challenges included the need for specialized diagnostic tools (radiography, ultrasonography, and laryngoscopy) and skilled personnel for safe handling and surgical intervention in camels, which may not be readily available in field settings. Future studies should include larger, multicenter investigations to better define epidemiology and risk factors. Incorporation of molecular diagnostics and fungal culture is recommended to confirm etiological agents and guide targeted therapy.

## Conclusion

This study documented four distinct upper respiratory and oropharyngeal conditions in camels: epulis (osteolipoma with osseous metaplasia), tonsillitis, and sialadenitis associated with entrapped dulla, and mycotic granulomatous laryngitis. These disorders were characterized clinically by varying degrees of oral or laryngeal obstruction, dysphagia, respiratory distress, neck stiffness, and reduced appetite. Hematological and biochemical analyses consistently revealed anemia, inflammatory leukogram changes (particularly neutrophilia and/or eosinophilia), and metabolic alterations, including electrolyte imbalances, reflecting systemic inflammatory and stress responses. Diagnostic imaging (radiography and ultrasonography), together with laryngoscopic examination, proved essential for identifying the location, extent, and nature of the masses. Histopathological examination confirmed the definitive diagnoses, including osteolipoma with osseous metaplasia and fungal pyogranulomatous inflammation consistent with *Aspergillus* spp. infection. Surgical excision of the affected dulla or abnormal masses resulted in a complete functional recovery in all cases within 6 months of follow-up. Favorable outcomes were strongly associated with early diagnosis, prompt surgical intervention, and appropriate postoperative care. Therefore, a comprehensive diagnostic approach combined with timely-applied surgical management represents an effective strategy for treating oropharyngeal and laryngeal disorders in camels.

## Data Availability

The datasets presented in this study can be found in online repositories. The names of the repository/repositories and accession number(s) can be found in the article/supplementary material.
